# Associations of the Alpha-Actinin Three Genotype with Bone and Muscle Mass Loss among Middle-Aged and Older Adults

**DOI:** 10.3390/jcm11206172

**Published:** 2022-10-19

**Authors:** Yoshiaki Taniguchi, Hyuma Makizako, Yuki Nakai, Yuto Kiuchi, Shoma Akaida, Mana Tateishi, Toshihiko Takenaka, Takuro Kubozono, Mitsuru Ohishi

**Affiliations:** 1 Graduate School of Health Sciences, Kagoshima University, Kagoshima 890-8544, Japan; 2 Department of Physical Therapy, Kagoshima Medical Professional College, Kagoshima 891-0133, Japan; 3 Department of Physical Therapy, School of Health Sciences, Faculty of Medicine, Kagoshima University, Kagoshima 890-8544, Japan; 4 Department of Mechanical Systems Engineering, Faculty of Engineering, Daiichi Institute of Technology, Kagoshima 899-4395, Japan; 5 Section for Health Promotion, Department of Preventive Gerontology, Center for Gerontology and Social Science, National Center for Geriatrics and Gerontology, Aichi 474-8511, Japan; 6 Tarumizu Municipal Medical Center Tarumizu Chuo Hospital, Kagoshima 891-2124, Japan; 7 Department of Cardiovascular Medicine and Hypertension, Graduate School of Medical and Dental Sciences, Kagoshima University, Kagoshima 890-8520, Japan

**Keywords:** ACTN3, gene, osteoporosis, sarcopenia, osteosarcopenia

## Abstract

Bone and muscle mass loss are known to occur simultaneously. The alpha-actinin three (*ACTN3*) genotype has been shown to potentially affect bone and muscle mass. In this study, we investigated the association between the *ACTN3* genotype and bone and muscle mass loss in community-dwelling adults aged ≥ 60 years. This study was a cross-sectional analysis of data from 295 participants who participated in a community health checkup. The *ACTN3* genotypes were classified as RR, RX, or XX types. Bone mass loss was defined as a calcaneal speed of sound T-score of <−1.32 and <−1.37, and muscle mass loss was defined as an appendicular skeletal muscle index of <7.0 kg/m^2^ and <5.7 kg/m^2^ in men and women, respectively. The percentages of XX, RX, and RR in the combined bone and muscle mass loss group were 33.8%, 30.8%, and 16.7%, respectively, with a significantly higher trend for XX. Multinomial logistic regression analysis showed that XX had an odds ratio of 3.00 (95% confidence interval 1.05–8.54) of being in the combined bone and muscle mass loss group compared to the RR group (covariates: age, sex, grip strength, and medications). The *ACTN3* genotype of XX is associated with a higher rate of comorbid bone and muscle mass loss. Therefore, *ACTN3* genotyping should be considered for preventing combined bone and muscle mass loss.

## 1. Introduction

The world’s population is aging rapidly, with the number of people aged 60 years and above projected to reach 1 billion by 2020 and 2.1 billion by 2050 [1]. Falls and fall-related fractures are common in the older population. One in three older adults falls each year [2], and approximately 10 percent of falls result in serious injuries [3]. In particular, hip fractures are often caused by falls, increasing the mortality rate and the need for care [4]. Although many factors contribute to falls and fractures, the loss of bone and muscle mass markedly increases the risk. In addition, bone and muscle mass loss are correlated [5,6,7]. Bone and muscle affect each other, with both mechanical stress and biochemical interactions thought to be involved [8]. A decrease in mechanical stress increases sclerostin and promotes bone resorption, whereas an increase in mechanical stress promotes bone formation [9]. Mechanical stress has been reported to increase protein synthesis and cause muscle hypertrophy [10]. Furthermore, bone and muscle have endocrine functions, with bone secreting osteokines and muscle secreting myokines, which enhance each other [11]. Recently, osteosarcopenia was defined as combined bone mass loss and sarcopenia [12,13,14]. The prevalence of osteosarcopenia has been reported to vary from 10.4% to 40.0% in different countries [15,16,17]. A meta-analysis showed that osteosarcopenia is associated with a significantly higher risk of falls, fractures, and mortality [18]. Preventing osteosarcopenia is important and requires investigation of the factors affecting both bone and muscle.

Some of the factors that affect bone and muscle mass include aging, physical inactivity, and vitamin D deficiency [19]. Bone mass and muscle mass are strongly influenced by genetic factors, with 60 to 70% being hereditary [20]. Alpha-actinin three (ACTN3) is a gene that affects bone and muscle mass [21]. ACTN3 is an actin-binding protein, and ACTN2 and 3 are major components of the z line in sarcomeres [22]. ACTN2 is expressed in all muscles, whereas ACTN3 is expressed only in Type II fibers [23,24]. North et al. identified a common stop-codon polymorphism (rs1815739; R577X) in *ACTN3* [23]. Individuals homozygous for the X allele do not express ACTN3 in Type II muscle fibers, in contrast to individuals with the RX or RR genotypes. The XX genotype has fewer Type II fibers [24]. In addition, ACTN3 is expressed in osteoprogenitor cells [25]. Loss of ACTN3 may affect bone mass.

Studies examining *ACTN3* genotypes and muscle mass have shown that individuals with XX and RX genotypes have less thigh muscle volume than those with the RR genotype [26]. In addition, a study involving older Korean adults showed that those with the XX genotype had lower skeletal muscle mass in the extremities than those with the RR genotype [27]. Studies examining the *ACTN3* genotype and bone mass have shown that the XX and RX genotypes have lower bone mass than the RR genotype [28]. Thus, if the *ACTN3* genotype is XX or RX, bone and muscle mass losses may occur simultaneously. This simultaneous loss is expected to increase the risk of falls and fractures. Previous studies have examined bone and muscle mass loss separately. However, these studies did not examine the association between comorbid bone and muscle mass loss and the *ACTN3* genotype. We hypothesized that *ACTN3* genotypes XX and RX would lead to concomitant loss of bone mass and muscle mass, as previously reported. The Asian Working Group for Sarcopenia defines older age as ≥60 or ≥65 years [29]. Menopause often occurs between the ages of 40 and 58 years, and bone mass density declines significantly after menopause [30]. This study aimed to determine the association between the *ACTN3* genotype and comorbid bone and muscle mass loss in community-dwelling adults aged ≥60 years. If the *ACTN3* genotype is associated with comorbid bone mass loss and muscle mass loss, *ACTN3* genotyping could be used to provide early prevention and intervention for those at a high risk for falls and fractures.

## 2. Materials and Methods

### 2.1. Study Participants

This cross-sectional study used data from the Tarumizu Study 2018 and 2019 participants who consented to undergo *ACTN3* genotyping. The Tarumizu Study is a community-based health check survey conducted jointly with Kagoshima University (Faculty of Medicine), Tarumizu City Office, and Tarumizu Chuo Hospital. The Tarumizu Study was open to citizens aged >40 years living in Tarumizu City by mailing a reply card. The surveys were conducted between July and December 2018 and June and December 2019. Among the Tarumizu Study 2018 and 2019 participants, 320 who were ≥60 and consented to *ACTN3* genotyping were included in this study. Exclusion criteria were defined as individuals with missing data for bone mass (*n* = 12), muscle mass (*n* = 3), or walking speed (*n* = 1), and those with a history of stroke (*n* = 9). The final number of participants in the analysis was 295 (mean age 73.2 ± 6.5 years, females 63.4%). This study was approved by the Kagoshima University (Faculty of Medicine) Ethics Committee (Ref No. 170351, 190319), and informed consent was obtained from all participants prior to their inclusion in the study.

### 2.2. ACTN3 Genotyping

The *ACTN3* genotype (rs1815739) was determined by collecting oral mucosal samples [26], and a DNA exercise-exercise gene test kit (Hersires International, Hiroshima, Japan) was used to analyze the samples. We ensured that participants fasted for 30 min prior to the test. Participants were asked to gargle with water and then use a sterile cotton swab (Tomy Works, Sakai, Japan) to rub the inside of their cheek for 1 min. EBS (Hiroshima, Japan) was used to extract DNA from the oral mucosa and determine the *ACTN3* genotype. A Mag Max DNA Multi-Sample Ultra Kit (Thermo Fisher Scientific, Paisley, UK) and King Fisher Flex Purification System (Thermo Fisher Scientific were used to extract genomic DNA from the swabs. The *ACTN3* genotype (rs1815739) was analyzed by polymerase chain reaction (PCR) with two pairs of primers (PCR-CTPP) using the KAPA2G Robust PCR Kit (Kapa Biosystems, Wilmington, MA, USA). *ACTN3* genotypes were classified as XX, RX, and RR.

### 2.3. Measurement of Bone Mass

Bone mass was measured using quantitative ultrasound equipment, CM-200 (Furuno, Nishinomiya City, Japan) [31]. The measurement site was the right calcaneus, and we confirmed the absence of calcaneal fracture. Quality control and calibration were undertaken before measurements were performed. Participants placed their right foot on a foot-pad adjusted to the size of their foot, and two transducers were placed on either side of their heel. Sound waves were then transmitted through the calcaneus from one transducer and received by the other. The signal was transmitted to a computer for processing, display, and storage [32]. The device uses the speed of sound (SOS) as a parameter to assess the health of participants’ bones. In this study, the T-score generated from the SOS was used to classify bone health. The T-score values obtained were based on a Japanese population survey provided by the manufacturer. Bone mass loss was defined as T-score <−1.32 for men and <−1.37 for women, based on a previous study [31].

### 2.4. Measurement of Muscle Mass

We assessed appendicular skeletal muscle mass using multifrequency bioelectrical impedance analysis with the InBody 470 (InBody Japan, Tokyo, Japan) [33]. The InBody 470 analyzer adopts a tetrapolar, eight-point tactile electrode system that separately measures the impedance of the arms, trunk, and legs at three different frequencies (5, 50, and 250 kHz) for each segment. A surface of the hand electrode was placed in contact with each of the five fingers, while the participants’ heels and forefeet were placed on a circular foot electrode. Appendicular skeletal muscle index (ASMI; kg/m^2^) was calculated by dividing limb skeletal muscle mass by the square of height. We defined muscle mass loss as ASMI <7.0 kg/m^2^ in men and <5.7 kg/m^2^ in women based on the Asian Working Group for Sarcopenia 2019 criteria [29].

### 2.5. Physical Performance

Physical performance was assessed using grip strength and walking speed. A Smedley-type handheld dynamometer (Grip-D; Takei Ltd., Niigata, Japan) was used to determine the maximum grip strength of the participants’ dominant hand [34]. Participants were asked to walk 14 m (divided into two 2 m long end zones and a 10 m long middle zone) at their usual walking speed to calculate their walking speed (m/s) [35]. The measurement was performed using a photoelectric sensor-type measuring device (YW; Yagami Inc., Aichi, Japan) to automatically measure walking time.

### 2.6. Sociodemographic Characteristics

The following sociodemographic characteristics were investigated: age (years), sex, body mass index (BMI), medications (*n*/day), use of osteoporosis medications, medical history, Geriatric Depression Scale 15 (GDS15) score, fall history (last year), and exercise habits (at least twice a week). Medications, use of osteoporosis medications, and medical history were assessed by licensed doctors or nurses through an interview.

### 2.7. Statistical Analysis

The characteristics of the three groups of *ACTN3* genotypes were compared using one-way analysis of variance for age, BMI, medications, GDS15, grip strength, and walking speed. Sex, osteoporosis medication, medical history, fall history, exercise habits, bone mass loss, and muscle mass loss were compared using the Mantel–Haenszel test for trend. Participants were classified into the following four groups based on bone and muscle mass cutoff values; normal, bone mass loss, muscle mass loss, and combined bone and muscle mass loss. The proportions of all groups in the three *ACTN3* genotypes were analyzed using the Mantel–Haenszel test for trend. A multinomial logistic regression analysis was performed with the four groups divided by bone mass and muscle mass as the dependent variables and *ACTN3* genotype as the independent variable. Age, sex, grip strength, and medications were included as covariates. IBM SPSS statistical software, version 27 (IBM Corp., Armonk, NY, USA) was used for data analysis. Statistical significance was set at *p* < 0.05.

## 3. Results

The characteristics of the *ACTN3* genotype are listed in Table 1. The variables that showed significant differences among the three groups were muscle mass loss (*p* = 0.006) and grip strength (*p* = 0.015), with a higher percentage of muscle mass loss in the XX genotype group. Bone mass loss was not significantly different, and there were no other significant variables among the *ACTN3* genotypes.

**Figure 1:**
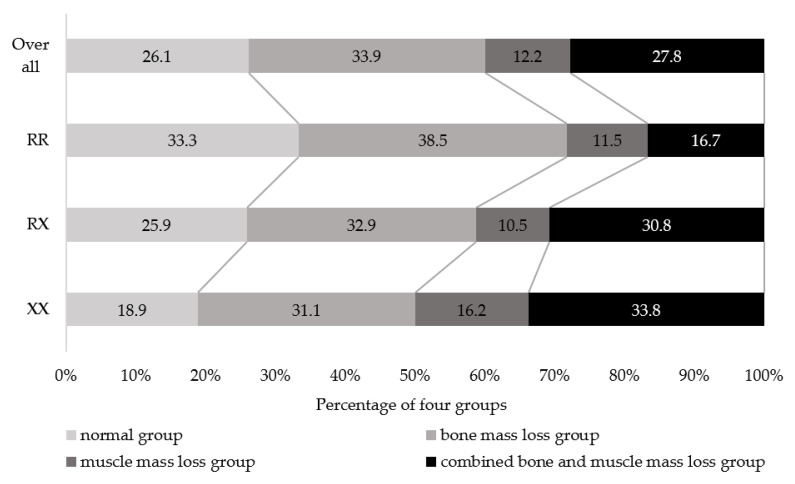
Percentage of the four groups classified according to bone and muscle mass in *ACTN3* genotypes. Mantel–Haenszel test for trend, *p* = 0.004.

Figure 1 shows the percentages of normal, bone mass loss, muscle mass loss, and combined bone and muscle mass loss in the three *ACTN3* genotypes. Overall percentages of normal, bone mass loss, muscle mass loss, and combined bone and muscle mass loss groups were 26.1, 33.9, 12.2, and 27.8%, respectively. Significant trend differences were observed in the percentages of all *ACTN3* genotypes (*p* = 0.004). The percentage of participants in the normal group was 18.9%, 25.9%, and 33.3% for XX, RX, and RR, respectively, with RR having the highest percentage. The percentage of combined bone and muscle mass loss was 33.8%, 30.8%, and 16.7% for XX, RX, and RR, respectively, and was the highest in XX.

Table 2 presents the results of the multinomial logistic regression analysis. The odds ratio of the combined bone and muscle mass loss group was significantly higher at 3.00 (95% confidence interval 1.05–8.54) than that for the normal group in XX, using RR as the reference. Bone mass loss and muscle mass loss groups were not associated with the *ACTN3* genotype.

## 4. Discussion

This is the first study to examine the coexistence of bone and muscle mass loss with the *ACTN3* genotype in community-dwelling adults aged ≥60 years. The results of this study suggest that bone and muscle mass loss are more likely to coexist in individuals with the XX genotype than in those with the RR genotype. However, there was no association between bone or muscle mass loss alone and the *ACTN3* genotype. Thus, the *ACTN3* genotype XX may be associated with combined bone and muscle mass loss.

In this study, the proportion of *ACTN3* genotypes was 25.1% for XX, 48.5% for RX, and 26.4% for RR. In general, the proportions of *ACTN3* genotypes in the Japanese population were 21.1–26.5% for type XX, 50.0–52.4% for type RX, and 20.3–26.9% for type RR [36,37,38,39], which are similar to the percentages of *ACTN3* genotypes in this study. Thus, the participants of this study can be considered representative of the general Japanese population. In addition, the percentage of older adults with comorbid bone and muscle mass loss in previous studies has ranged from 21.4 to 27.9% [17,40]. The overall proportion of participants with combined bone and muscle mass loss in this study was 27.8%, similar to that reported in previous studies. Interestingly, this study indicated a higher percentage of combined bone and muscle mass loss in the XX genotype (33.8%) than that in the RR genotype (16.7%). Therefore, loss of bone and muscle mass may be more likely to coexist in the XX genotype than in the RR genotype. Conversely, those with the *ACTN3* genotype RR had a higher percentage of stable combined bone and muscle mass at 33.3% compared with 18.9% in the XX genotype. A meta-analysis showed that RR of the *ACTN3* genotype is more common in sprint and power athletes [41]. In addition, 12 weeks of resistance training in older adults showed that RR increased maximal strength by approximately 30% on bench press and 15% on leg extension compared to those in XX [42]. Bone mass is higher in RR than that in XX [25], and the percentage of people without osteoporosis who have reduced muscle mass is approximately 20% lower than that of those with osteoporosis [43]. The *ACTN3* genotype RR may be protective against bone and muscle mass loss.

There are many common factors that cause bone and muscle mass loss, including sex hormone deficiencies, lifestyle factors such as inactivity and smoking, and comorbidities, such as genetic factors [44]. ACTN3 plays an important role in regulating protein synthesis and degradation signaling in the skeletal muscle and has been shown to affect muscle mass from early after birth [45]. Furthermore, ACTN3 knockout mice have been shown to have decreased bone mass due to decreased osteoblast activity and increased osteoclast activity [25]. In addition, the XX genotype may have increased osteocalcin, procollagen 1 N-terminal propeptide (P1NP), and β-isomerized C-terminal telopeptide (β-CTx) activity, indicating high bone metabolic turnover [46]. The *ACTN3* genotype has a direct effect on the bone and muscle and is likely to coexist with bone and muscle mass loss. This study did not find an association between the *ACTN3* genotype and bone mass or muscle mass loss alone. This may have been influenced by factors related to bone and muscle mass loss singly. Factors associated with bone mass loss include a history of fragility fractures and maternal hip fractures, whereas factors associated with muscle mass loss include low albumin levels, angiotensin-converting enzyme inhibitor use, and dyslipidemia [47]. However, these factors were not investigated, which is a limitation of this study. Additionally, several genes other than *ACTN3* are active in both bone and muscles [48].

There are several limitations of our study that should be considered. As this was a cross-sectional study, it is unclear whether the *ACTN3* genotype directly affects bone and muscle mass loss. We cannot discount the possibility that one occurs first and the other is affected by its decline. In addition, the severity and duration of bone and muscle mass loss were not investigated and should be included in future research. The participants in this study were respondents in a health check, who consented to *ACTN3* genotyping, and were not randomly selected. Additionally, a sample size estimate was not calculated. In measuring bone mass, this study used SOS from quantitative ultrasound and not dual-energy X-ray absorptiometry, which is the gold standard for osteoporosis diagnosis. Furthermore, other potential covariates related to bone and muscle mass, such as nutrition, lifestyle, hormonal factors, physical activity, and other genes, remain to be considered. Bone and muscle mass are affected by sex, but the number of participants in this study was not sufficient to allow separate analysis by sex. Future studies should include a longitudinal design on a larger number of participants and examining the *ACTN3* genotype and its effect on combined bone and muscle mass loss according to sex.

## 5. Conclusions

This study examined the association between the *ACTN3* genotype and bone and muscle mass loss in community-dwelling older adults aged ≥60 years. Participants with *ACTN3* genotype XX were more likely to have comorbid bone and muscle mass loss. Although various factors are involved in bone and muscle mass loss, the *ACTN3* genotype is thought to be a major contributing factor. Combined bone and muscle mass loss may increase the risk of falls and fractures and may necessitate preventive measures for those with the XX genotype. Older adults with *ACTN3* genotype XX may have combined bone and muscle mass loss, and exercise approaches and treatment strategies should be considered according to the *ACTN3* genotype.

## Figures and Tables

**Table 1:** Characteristics of Participants by *ACTN3* Genotype, Mean ± SD, or %.

		Genotype			
Variable	Overall (*n* = 295)	XX (*n* = 74, 25.1%)	RX (*n* = 143, 48.5%)	RR (*n* = 78, 26.4%)	*p*
Age, years	73.2 ± 6.5	73.9 ± 6.9	73.0 ± 6.9	72.8 ± 5.3	0.500
Female, *n* (%)	187 (63.4)	46 (62.2)	102 (71.3)	39 (50.0)	0.109
BMI, kg/m^2^	22.8 ± 3.1	22.2 ± 3.2	22.9 ± 3.2	23.2 ± 2.8	0.122
Medications, number/day	3.0 ± 3.0	3.7 ± 3.3	2.9 ± 3.0	2.5 ± 2.5	0.054
Osteoporosis medications, *n* (%)	46 (15.6)	11 (14.9)	27 (18.9)	8 (10.3)	0.419
Medical history, *n* (%)					
Heart disease	23 (7.8)	6 (7.7)	11 (7.7)	6 (8.1)	0.925
Diabetes mellitus	36 (12.2)	10 (13.5)	16 (11.2)	10 (12.8)	0.904
Osteoporosis	55 (18.6)	15 (20.3)	32 (22.4)	8 (10.3)	0.107
Thyroid disease	22 (7.5)	1 (1.4)	17 (11.9)	4 (5.1)	0.405
Respiratory disease	29 (9.8)	10 (13.5)	14 (9.8)	5 (6.4)	0.142
Malignant tumor	33 (11.2)	6 (8.1)	19 (13.3)	8 (10.3)	0.690
GDS-15, score	2.6 ± 2.4	2.9 ± 2.6	2.7 ± 2.5	2.4 ± 2.1	0.397
Fall history, *n* (%)	45 (15.3)	13 (17.6)	22 (15.4)	10 (12.8)	0.416
Exercise habit, *n* (%)	237 (80.3)	61 (82.4)	112 (78.3)	64 (82.1)	0.965
Muscle mass loss, *n* (%)	118 (40.0)	37 (50.0)	59 (41.3)	22 (28.2)	0.006
Bone mass loss, *n* (%)	182 (61.7)	48 (64.9)	91 (63.6)	43 (55.1)	0.213
Grip strength, kg	25.7 ± 7.9	25.3 ± 7.2	24.8 ± 7.8	27.9 ± 8.4	0.015
Walking speed, m/s	1.3 ± 0.2	1.3 ± 0.2	1.3 ± 0.2	1.3 ± 0.2	0.979

BMI, body mass index; GDS-15, Geriatric Depression Scale 15; SD, standard deviation. One-way analysis of variance was used for continuous measures and percentage using the Mantel–Haenszel test for trend.

**Table 2:** Odds ratio of the A*CTN3* genotype in four groups classified by bone mass and muscle mass.

	Normal Group vs		
Independent Variable	Bone Mass Loss Group OR (95% CI)	Muscle Mass Loss Group OR (95% CI)	Combined Bone and Muscle Mass Loss Group OR (95% CI)
RR	reference	reference	reference
RX	0.75 (0.35–1.60)	1.03 (0.37–2.88)	1.62 (0.66–3.98)
XX	1.14 (0.45–2.89)	2.30 (0.73–7.30)	3.00 (1.05–8.54) *

Covariates: age, sex, grip strength, medications. OR, odds ratio; CI, confidence interval. * *p* = 0.039.

## Data Availability

There are no linked research datasets for this study. The authors do not have permission to share the data.
